# Myeloid neoplasm post cytotoxic treatment in patients with multiple myeloma

**DOI:** 10.1002/jha2.1017

**Published:** 2024-09-30

**Authors:** Ke Xu, Eleanor Kaffo, Robert Baker, Elisabeth Nacheva, Annabel McMillan, Lydia Lee, Xenofon Papanikolaou, Rakesh Popat, Jonathan Sive, Kwee Yong, Neil Rabin, Charalampia Kyriakou, Rajeev Gupta

**Affiliations:** ^1^ Department of Haematology, University College London Hospital NHS Foundation Trust University College London London UK; ^2^ Specialist Integrated Haematology Malignancy Diagnostic Service, Health Services Laboratories University College London Hospitals NHS Foundation Trust, University College London London UK; ^3^ UCL Medical School University College London London UK; ^4^ UCL School of Life and Medical Sciences, University College London London UK

Dear Editor,

Myeloma and monoclonal gammopathy of undetermined significance patients are at higher risk of myelodysplastic syndrome (MDS)/acute myeloid leukaemia (AML) [1]. Cytotoxic treatment, including alkylating agents such as high‐dose melphalan (HDM), further increases their risk of post cytotoxic treatment‐myeloid neoplasm (pCT‐MN) [2]. HDM followed by autologous stem cell transplantation (ASCT) prolongs progression‐free survival. It is the standard of care in the UK for transplant‐eligible patients. Our centre performs 150 HDM ASCT every year. The study aimed to review pCT‐MN cases among patients with multiple myeloma in our centre and describe their characteristics, cytogenetics and molecular risk, treatment regimen and outcome.

We retrospectively reviewed all new pCT‐MN cases (as defined by WHO 5th edition classification) with background myeloma, whose pCT‐MN was diagnosed and treated at our centre's specialist integrated haematological malignancy diagnostic service. The data cutoff date was 30 June 2024. Our standard diagnostic MDS/AML fluorescence in situ hybridization (FISH) panel consists of break apart or fusion probes targeting *KMT2A*, *CBFB::MYH11* inv(16), *RUNX1T1::RUNX1* t(8;21), *PML::RARA* t(15:17) and *MECOM*, and probes targeting 5q, 7q and 17p (Cytocell). Molecular karyotyping (8 × 60K oligonucleotide arrays, Agilent) was used to assess copy number variations across the whole genome. Targeted myeloid NGS panel analysis (Table S1) was used according to the manufacturer's instructions to detect pathogenic variants.

A total of 906 patients with multiple myeloma were actively followed up at our centre between 1 January 2018 and 31 December 2022. They were diagnosed with myeloma between 2004 and 2022. The median age of myeloma diagnosis was 60 years (range: 28–93 years). Six pCT‐MN patients with multiple myeloma were identified. Their characteristics are summarised in Table 1. All six patients had previous alkylator therapy and presented with progressive cytopenia. Five were male and one was female. The median age of symptomatic myeloma diagnosis was 66 years (range: 58–77 years). Four were standard risk on CD138‐cell FISH. Two had no FISH result. All five transplant‐eligible patients had HDM ASCT. Other myeloma treatments they received were bortezomib thalidomide and dexamethasone (VTD), ixazomib lenalidomide and dexamethasone (IRD), lenalidomide and dexamethasone (RD), daratumumab, CC220, bortezomib lenalidomide and dexamethasone (VRD), cyclophosphamide lenalidomide and dexamethasone (CRD), isatuximab pomalidomide and dexamethasone (IsaPD), bortezomib cyclophosphamide and dexamethasone (VCD) and PD. The median lines of myeloma treatment received were two (range:1–6). The median time from diagnosis of myeloma to diagnosis of pCT‐MN was 83 months (range: 18–233 months). Two patients were diagnosed with AML, two with MDS‐excess of blast (EB) and two with MDS‐multilineage dysplasia (MLD). One AML was 2022 European LeukemiaNet (ELN) [3] intermediate risk, one AML was ELN adverse risk, three MDS were Revised International Prognostic Scoring System (IPSS‐R) very high‐risk and one MDS was IPSS‐R high risk. The cytogenetic abnormalities detected were deletion 5q (3/6), deletion 7q (2/6), monosomy 7 (2/6), *MECOM* rearrangement (1/6), 6p chromothripsis (1/6), iAMP21(1/6) and gain *RUNX1* (2/6). The pathogenic variants detected by NGS were *TP53* (2/6), *RUNX1* (2/6), *AXSL1* (2/6), *DNMT3A* (1/6)*, PTPN11* (1/6)*, KRAS* (1/6) and *ETV6* (1/6). The treatment received for pCT‐MN was azacytidine alone (4/6) or venetoclax with azacytidine (1/6). None of the patients were fit for an allograft stem cell transplant. None of the patients had achieved any cytogenetic or molecular complete response. At the last follow‐up, one patient was still alive. The median overall survival from a pCT‐MN diagnosis was 10 months.

**TABLE 1:** Characteristics of six myeloma patients who developed post cytotoxic treatment‐myeloid neoplasm (pCT‐MN).

**Patient**	**1**	**2**	**3**	**4**	**5**	**6**
**Age at myeloma diagnosis (years)**	66	66	58	77	70	58
**Age at ASCT (years)**	67	67	59	NA	72	59 (1^st^ ASCT) 64 (2^nd^ ASCT)
**Age at pCT‐MN diagnosis (years)**	70	67	67	81	80	77
**Sex**	M	M	M	M	F	M
**CD138 cell FISH at diagnosis**	Normal	Normal	No result	Standard risk	Normal	No result
**ASCT**	Yes	Yes	Yes	No	Yes	Yes
**Treatment regimens**	VTD, HDM ASCT IRD	VTD, HDM ASCT	RD, HDM ASCT, Velcade maintenance ITD VRD CRD IsaPD	CVD CRD	CRD VCD RD PD, HDM ASCT Daratumumab CC220	Unknown, HDM ASCT Unknown,2^nd^ HDM ASCT
**Lines of MM treatment**	2	1	5	2	6	2
**MM treatment response at diagnosis of pCT‐MN**	VGPR	PR	SD	progression	PR	CR
**Time from MM diagnosis to pCT‐MN diagnosis (months)**	45	18	120	49	117	233
**pCT‐MN diagnosis**	MDS‐MLD	AML	MDS‐EB2	MDS‐EB2	AML	MDS‐MLD
**pCT‐MN risk stratification**	IPSS‐R very high risk	ELN adverse risk	IPSS‐R very high risk	IPSS‐R very high risk	ELN intermediate risk	IPSS‐R high risk
**MDS/AML FISH**	del 5q31 −7	*MECOM* rearrangement 7q loss	7q del +21	5q del iAMP21 6p chromothripsis	Gain of *RUNX1*	del5q −7
**Molecular karyotyping**	5p15/p14 loss, 5q21/q34 loss, −7, 12p13/p12 del	‐ 7	del 7q, gain 21q2 (*RUNX1*)	5q11q33, 6p21‐q11q15 losses, 6p chromothripsis, −18, +21, 21q11q22 amp (incl. *RUNX1*), ETS amp	Normal	No result
**Pathogenic variants by myeloid NGS**	*TP53*	*ASXL1* *RUNX1*	*RUNX1* *DNMT3A* *PTPN11* *KRAS*	*ASXL1*	*ETV6*	*TP53*
**pCT treatment**	Azacitidine	Venetoclax+Azacitidine	Azacitidine	Supportive care	Azacitidine	Azacitidine
**Transformed to AML**	Yes	NA	Yes	No	NA	Yes
**Time to transform to AML (months)**	12	NA	3	NA	NA	6
**Alive or deceased**	D	D	D	D	A	D
**Follow up time from pCT‐MN diagnosis (months)**	13	8	4	7	12	12

Abbreviations: AML, acute myeloid leukaemia; ASCT, autograft stem cell transplant; CR, complete remission; CRD, cyclophosphamide lenalidomide and dexamethasone; ELN, European LeukemiaNet; FISH, fluorescence in situ hybridization; HDM, high‐dose melphalan; ITD, ixazomib thalidomide and dexamethasone; IRD, ixazomib lenalidomide and dexamethasone; IsaPD, isatuximab pomalidomide and dexamethasone; MDS‐MLD, myelodysplastic syndrome‐multilineage dysplasia; NA, not applicable; pCT‐MN, post cytotoxic treatment‐myeloid neoplasm; PR, partial response; RD, lenalidomide and dexamethasone; SD, stable disease; VCD, bortezomib cyclophosphamide and dexamethasone; VGPR, very good partial response; VRD, bortezomib lenalidomide and dexamethasone; VTD, bortezomib thalidomide and dexamethasone.

pCT‐MN commonly occurs 5–10 years after exposure to alkylating agents. It is commonly associated with chromosome 5 and 7 loss. About 20%–30% of pCT‐MN patients have balanced chromosomal translocations. They are associated with a short latency period, often present as overt AML without a preceding MDS phase [4]. The prognosis of pCT‐MN is generally poor. Cases associated with chromosome 5 and 7 abnormalities and a complex karyotype have a particularly poor outcome, with a median survival time of less than 1 year [4]. As myeloma patients live longer, the incidence of late complications of therapy, such as pCT‐MN, increases. The published cumulative pCT‐MN incidence in myeloma patients is 0.3%–12.2% [5]. Clonal haematopoiesis was reported in a case–control study by Takahashi et al. as a risk factor for pCT‐MN in solid tumour and lymphoma patients [6]. In a single–centre retrospective study by Mouhieddine TH et al., clonal haematopoiesis of indeterminate potential (CHIP) was found in 21.6% (136/629) of pre‐ASCT myeloma patients’ stem cell product with infrequent (2.9%) *TP53*. However, CHIP was not found to be predictive of pCT‐MN in myeloma patients treated with ASCT [7]. Nadiminti et al. found that lenalidomide exposure was associated with a significantly higher risk of pCT‐MN [8]. Further prospective studies are needed to identify risk factors that predict pCT‐MN.

In our cohort of patients with multiple myeloma, the cumulative incidence of pCT‐MN was 0.7%. The median time from diagnosis of myeloma to diagnosis of pCT‐MN was 83 months. Our cohort of patients with pCT‐MN had high‐risk cytogenetics or molecular features in whole bone marrow at pCT‐MN diagnosis and short overall survival. Our findings are consistent with previous publications [4, 5]. With increasingly effective novel therapies for myeloma, we should consider the risk of pCT‐MN when recommending ASCT to newly diagnosed patients, particularly in the context of standard‐risk disease and a deep response to induction.

## AUTHOR CONTRIBUTIONS

Ke Xu designed the study, analysed the data and wrote up the manuscript. Eleanor Kaffo analysed the data. All the authors critically revised the final version of the manuscript.

## CONFLICT OF INTEREST STATEMENT

The authors declare no conflicts of interest.

## FUNDING INFORMATION

The author(s) received no financial support for the research, authorship, and publication of this article.

## Supporting information



Supporting Information

## Data Availability

The datasets generated during and/or analysed during the current study are available from the corresponding author on request.
